# Effects of Waste Powders of Tuff Manufactured Sand on Characteristics of Highly Ductile Polyvinyl Alcohol Fiber Engineered Cementitious Composite

**DOI:** 10.3390/ma19020296

**Published:** 2026-01-12

**Authors:** Tao Liu, Youjia Wang, Bentian Yu, Shikai Ji, Kai Wang, Fangling Wang

**Affiliations:** 1 Power China Guiyang Engineering Corporation Limited, Guiyang 550081, China; liutaojianzhu_gyy@powerchina.cn; 2 Jiayuguan Track Maintenance Division, China Railway Lanzhou Group Co., Ltd., Lanzhou 730070, China; 15352112596@163.com; 3 School of Civil Engineering, Lanzhou Jiaotong University, Lanzhou 730070, China; 15991721565@163.com (S.J.); wangkai010152@163.com (K.W.); 18188511732@163.com (F.W.)

**Keywords:** tuff stone powder, engineered cementitious composites, mechanical properties, drying shrinkage, microstructure

## Abstract

**Highlights:**

**What are the main findings?**

- The mechanical properties of PVA-ECC are best when the content of tuff stone powder instead of quartz sand is 40%.
- The incorporate of tuff powder as a replacement for quartz sand can improve the hydration and reduce harmful pores in ECC.
- The incorporate of tuff powder as a replacement for quartz sand can improve the strain-harding characteristics of ECC.
- The dry shrinkage of ECC increase with increasing of tuff stone powder content and age.

**What are the implication of the main findings?**

- From the perspective of mix proportion optimization for ECC, the identified optimum replacement ratio of 40% for quartz sand with tuff powder provides a clear technical reference for designing high-performance polyvinyl alcohol fiber-reinforced ECC (PVA-ECC). This result substantiates that tuff powder is a viable and effective alternative to conventional quartz sand. Its incorporation enhances the mechanical performance of ECC, promotes cement hydration, and refines the pore structure by reducing the volume of harmful pores. From an engineering application standpoint, this substitution not only reduces reliance on non-renewable quartz sand resources but also contributes to lowering the production cost of ECC.
- The use of tuff powder as a substitute for quartz sand enhances the strain-hardening behavior of ECC, yet simultaneously intensifies its drying shrinkage with increasing replacement content and curing age. This trade-off is crucial for the rational design and practical application of tuff-modified ECC. The improved strain-hardening performance confirms that tuff powder is a high-performance alternative, enabling ECC to exhibit superior ductility and crack resistance. Consequently, future research should focus on developing synergistic optimization strategies to mitigate shrinkage-induced cracking while maintaining the beneficial strain-hardening characteristics.

**Abstract:**

In this paper, a highly ductile polyvinyl alcohol fiber engineered cementitious composite (PVA-ECC) was developed by replacing quartz sand (QS) with tuff stone powder (TP) at different replacement ratios of 20%, 40%, 60%, 80%, and 100%. The resulting mechanical properties and drying shrinkage were determined for the developed ECC. Qualitative and quantitative analyses of hydration products, pore structure, and micro-morphology of ECC were conducted by X-ray diffraction, thermogravimetric analysis, Fourier transform infrared spectroscopy, pore size and porosity, and scanning electron microscopic imaging. The influencing mechanism of tuff stone powder content on ECC performance was also studied at a micro level. It was found that with the increase in the replacement ratio of tuff stone powder, the ultimate tensile strain and tensile peak stress of ECC all exhibited an increasing trend, which declined afterward. The variation in compressive and flexural strengths also showed a similar pattern. When the replacement ratio of tuff stone powder was 40%, the ultimate tensile strain, peak tensile stress, flexural strength, and compressive strength were higher than the control group by 15.1%, 4.7%, 16.3%, and 10.7%, respectively. When the content of tuff stone powder did not exceed 80%, it could fill the internal pores of the ECC matrix, which reduced harmful pores. With the increase in tuff stone powder content, calcite content increases gradually while the Ca(OH)_2_ amount decreases. It can be seen that tuff stone powder can improve ECC hydration products. However, incorporating tuff stone powder does not produce new hydration products. Incorporating tuff stone powder increased the drying shrinkage of ECC, and the value of drying shrinkage increased with the increase in the replacement ratio of tuff stone powder.

## 1. Introduction

Ordinary concrete is prone to cracking and large crack widths, leading to the ingress of external harmful ions and moisture entering the interior of the matrix more easily, which severely affects the durability and safety of the structures [1,2,3]. In order to address the shortcomings of concrete, Li [4,5,6] proposed a high-ductility engineered cementitious composite (ECC), which exhibits multiple cracking and strain hardening under tensile and flexural loads. The distinctive characteristics of ECC are mainly due to the bridging effect. Once the matrix is fractured, the stress at the crack is borne by the fibers surrounding the matrix, which then transfer the stress to the surrounding uncracked matrix [7,8,9], making ECC exhibit excellent ductility and crack width control ability [10,11,12]. The traditional ECC incurs high production costs and contains high-toughness polyvinyl alcohol (PVA) fibers, quartz sand, cement, and various mineral admixtures [13,14]. ECC has been applied in several projects, and its capacity to control cracks and durability is significantly superior to ordinary concrete [15,16,17]. Nevertheless, the high cost of PVA fiber and superfine quartz sand hinders its large-scale applicability in practical engineering.

Researchers have started using inexpensive fine aggregate to prepare ECC and reduce its preparation cost. Li et al. [18,19] used river sand as a fine aggregate in preparing ECC and found that impurities in river sand lead to unsaturated cracks and reduce the tensile strain performance of ECC. In most studies, fine aggregate <0.3 mm particle size was used because of specific requirements regarding fine aggregate selection for traditional ECC. Using river sand to prepare ECC produces new construction waste and results in the utilization of natural resources. The cement industry accounts for a large proportion of global CO_2_ emissions, and reducing the carbon emissions of concrete raw materials is the primary way to reduce carbon emissions. Therefore, using low-carbon green materials is crucial for the sustainable development of ECC in the future [20,21,22].

In the production process of manufactured sand, approximately 10% of the stone powder with a particle size of <75 um is produced, and its particle size is similar to that of quartz sand used in ECC [23,24,25]. Therefore, for ECC cost reduction and efficient resource utilization, using stone powder as a fine aggregate in ECC has gained much attention. Ren et al. [26] used granite powder instead of quartz sand to prepare ECC and found improved compactness of the ECC matrix by optimizing the pore structure. Further, adding granite powder effectively enhanced the fiber–matrix interface and pull-out resistance of the composite, effectively improving the ductility. For 25% granite powder content, the mechanical properties of ECC were the best. Zhang et al. [27] found that replacing 100% quartz sand with granite powder led to optimal mechanical properties, which differs from the previous results. Maninder et al. [28,29,30,31] used slurry powder instead of micro quartz sand to prepare ECC and concluded that incorporating stone powder enhances the flexural strength of ECC. Turk et al. [32,33,34] used limestone powder instead of fine quartz sand to prepare ECC and found that fine limestone powder particles could effectively fill the excess voids between the cementitious materials, thereby enhancing the step-by-step filling effect of the ECC matrix. It also positively impacted the ductility and fiber dispersion, while the mechanical properties of ECC were optimal for 100% limestone powder dosage. Li [35] used granite porphyry powder (D90 = 269 μm), limestone powder (D90 = 20 μm), and quartz powder (D90 = 204 μm) instead of manufactured sand to prepare ECC, and the results showed that stone powder could enhance ECC strength.

**Figure 1:**
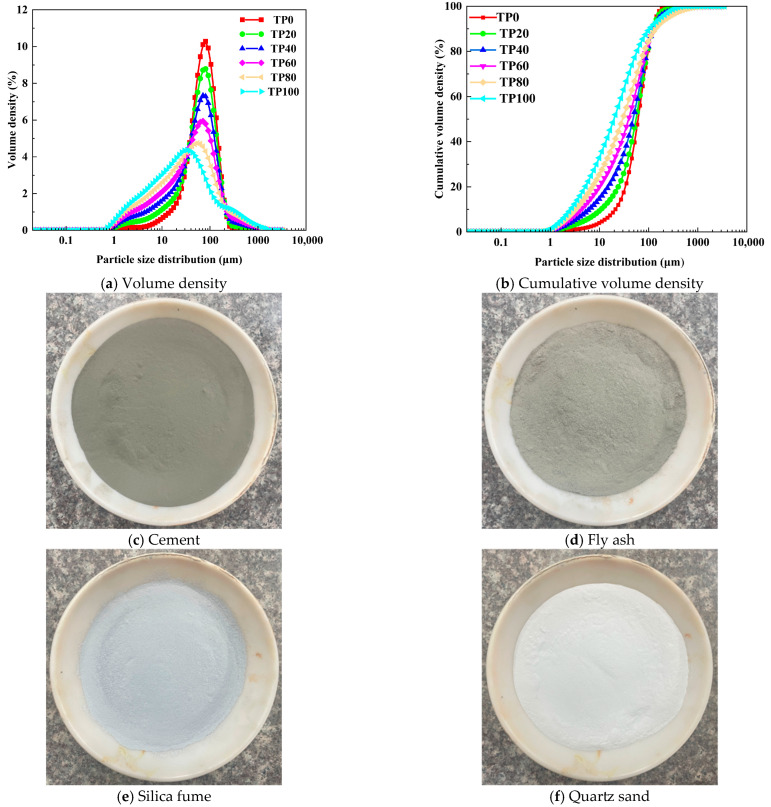

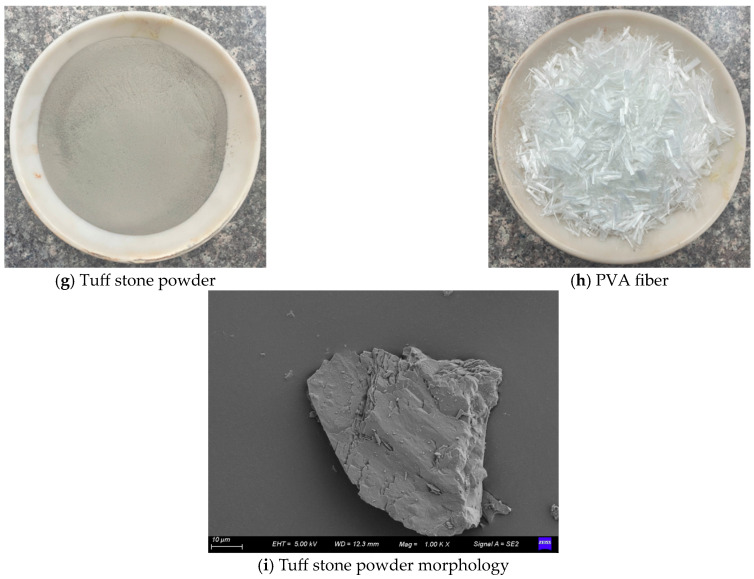
Raw materials.

In summary, current research on low-cost ECC preparation primarily focuses on substituting quartz sand with granite stone powder or limestone powder. However, systematic investigations into the utilization of tuff stone powder—especially the waste by-product derived from manufactured sand and gravel production—for ECC development remain scarce. Notably, tuff is the predominant raw material for manufactured sand and gravel production in the Gansu region, resulting in the generation of substantial volumes of waste tuff stone powder that urgently requires resourceful reuse. As illustrated in Figure 1, the particle size distribution curve reveals that tuff stone powder is finer and exhibits a broader particle size range compared to fine quartz sand. This unique characteristic enables it to more effectively fill the internal pores of the ECC matrix, thereby optimizing the matrix compactness. Furthermore, the gradation of tuff stone powder is highly compatible with that of quartz sand, laying a solid foundation for its potential as a direct quartz sand substitute in ECC. By leveraging this regionally abundant solid waste, this study not only addresses the resource utilization challenge of tuff stone powder but also pioneers a novel, cost-effective pathway for ECC fabrication—filling the existing research gap in applying tuff stone powder to ECC.

In this paper, ECC was developed using tuff stone powder with different replacement ratios instead of quartz sand. The resulting mechanical properties and drying shrinkage of ECC were determined, and the influencing mechanism of tuff stone powder on ECC was examined. Qualitative and quantitative analyses of hydration products, pore structure, and micro-morphology of ECC were conducted by X-ray diffraction (XRD), thermogravimetric analysis (TG), Fourier transform infrared spectroscopy (FTIR), pore size and porosity, and scanning electron microscopy (SEM). The influencing mechanism of tuff stone powder content on the ECC performance was explained at the micro level. The utilization of tuff stone powder in the preparation of ECC not only utilizes solid waste resources but also reduces the cost of ECC preparation, providing a basis for the rational application of tuff stone powder in the future.

## 2. Materials and Methods

### 2.1. Raw Materials

The cement used in the study was 42.5 grade ordinary Portland cement (P·O 42.5) produced by Gansu Qilianshan Cement Company (Lanzhou, China). The chemical and physical properties of cement are presented in Table 1 and Table 2, respectively. Class F II fly ash produced by Gansu Hongda Investment and Development Group Co., Ltd. (Lanzhou, China). was also used; its properties are given in Table 3. The SY-85 SD silica fume produced by Gansu Sanyuan Silicon Material Co., Ltd. (Lanzhou, China). was used as the silica fume. The properties are listed in Table 4. The superplasticizer was an HLX-type polycarboxylate superplasticizer produced by Shanxi Weike Building Materials Co., Ltd. (Changzhi, China). The tuff stone powder having the pozzolanic activity index < 65% was used in the study. It was a by-product of the sand and gravel produced by Gansu Jiantou Green Building Materials Industry Development Group Co., Ltd. (Lanzhou, China). The XRF and XRD analyses are tabulated in Table 5 and Table 6. PVA fibers (REC-12) produced by Kuraray Co., Ltd. (Tokyo, Japan) were also used. The giber properties are given in Table 7. 60 mesh quartz sand produced by Shanxi Junhong New Material Co., Ltd. (Taiyuan, China). was used as fine aggregate, while ordinary tap water was used for mixing the raw materials. The particle size distribution curve of fine aggregate is shown in Figure 1a,b, while the raw materials are shown in Figure 1c–h. The morphology of tuff stone powder is shown in Figure 1e.

### 2.2. Mix Proportion

In order to explore the influence of tuff stone powder on the tensile strength, compressive strength, flexural strength, drying shrinkage, and microstructure of PVA-ECC, the content of tuff stone powder was used as a variable in this experiment. Six specimen groups with varying tuff stone powder contents were designed to replace 0%, 20%, 40%, 60%, 80%, and 100% quartz sand, corresponding to TP0 ~ TP100, respectively. ECC has higher rheological requirements because the uniform dispersion of fibers requires appropriate viscosity and fluidity. It is a standard method to control the viscosity of fresh mortar by controlling the water-to-binder ratio [36,37,38]. Therefore, in this paper, the specific amount of superplasticizer was adjusted according to the same fluidity (160 mm–180 mm) range of the mixture to ensure the feasibility of the test. The water-to-binder ratio was 0.4, the sand-to-binder ratio was 0.65, and the volume content of PVA fiber was 2.5%. The specific mix proportions are detailed in Table 8.

### 2.3. Sample Preparation

**Figure 2:**
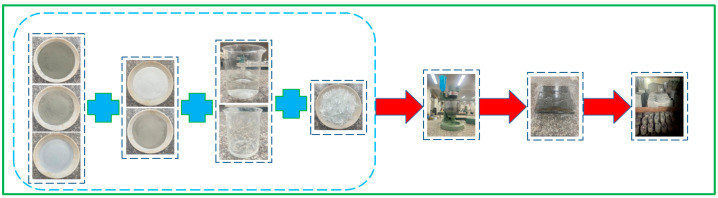
Sample preparation process.

The cementitious materials and fine aggregates were weighed and loaded into the mixing pot. After dry mixing for 120 s, water and water reducer were added and stirred for 1 min, then fibers were added slowly. After fiber addition, the mix was stirred rapidly for 60 s, and specimens were cast from the prepared mix. Due to the poor fluidity of ECC, it is necessary to fill the mold several times in layers. After filling, the molds were compacted on a vibrating table for 60 s. Subsequently, the surface of the cast specimens in the molds was covered with a cling film. Then, the specimen was placed in a standard curing room for 28 days after removing the mold for 24 h, and mechanical properties, X-ray diffraction (XRD), thermogravimetric analysis (TG), Fourier Transform infrared spectroscopy (FTIR), and scanning electron microscopy (SEM) tests were carried out afterward. The specimen preparation process is shown in Figure 2.

### 2.4. Experimental Methods

**Figure 3:**
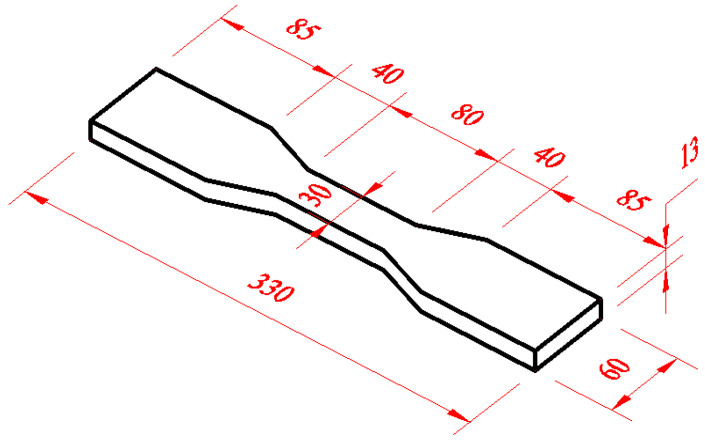
Tensile specimen model.

The uniaxial tensile test was conducted following the “Test Method for Mechanical Properties of Highly Ductile Fiber-Reinforced Cementitious Composites” (JC/T 2461-2018 [39]), and the dog bone-type specimen was used [40], with dimensions of 80 mm × 30 mm × 13 mm. Hengsi Shengda WDW-50 micro-computer control electronic universal testing machine purchased from Jinan Shidai Shantou Instrument Co. (Jinan, China) was used for loading; the loading speed was 0.05 mm/min, and the specimen size is shown in Figure 3. The flexural test was carried out under the “Cement Mastic Sand Strength Test Method” (ISO method) [41]. The size of the prism specimen was 40 mm × 40 mm × 160 mm, and the WHY-300/10 microcomputer-controlled compression tester produced by Shanghai Hualong Testing Instruments Co. (Shanghai, China) was used for testing. A 40 mm × 40 mm × 80 mm specimen was used for compressive strength testing using WHY-300/10 microcomputer-controlled pressure testing machine produced by Shanghai Hualong Testing Instrument Co.

The X-ray diffraction (XRD) was performed using the Rigaku benchtop XRD-MiniFlex600 instrument purchased from Hangzhou Leimei Technology Co. (Hangzhou, China). Small samples were taken from the center of the crushed specimen and stored in anhydrous ethanol. Before the test, the sample was oven-dried at 60 °C for 6 h. After drying, the sample to be tested was ground into a fine powder of <75 μm, and the phase analysis was performed in the scanning range of 2θ angle 5° ~80°.

Thermogravimetric analysis (TG) was performed using the NETZSCH TG 209 Analyzer purchased from German Naterix Instrument Manufacturing Co., Ltd. (Schelbe, Germany) to analyze the physical properties. The temperature range was 30–1000 °C, and the heating rate was 10 °C/min. Small samples were taken from the center of the crushed test block, soaked in anhydrous ethanol until the hydration reaction was terminated, oven-dried at 60 °C for 24 h, and finally ground into fine powder less than 75 μm for testing.

Fourier transform infrared spectroscopy (FTIR) test was performed using a VERTEX70 Fourier transform infrared spectrometer from Bruker (Karlsruhe, Germany). A small sample was taken from the center of the crushed specimen and stored in anhydrous ethanol. Before the test, the sample was placed in an oven at 60 °C for 6 h. After drying, the sample to be tested was mixed with KBr and ground into a fine powder smaller than 2 μm. After grinding, the microscopic qualitative analysis was performed.

The pore size and porosity test was carried out using Macro MR12-150H-I multi-functional nuclear magnetic resonance microstructure analysis and an imaging system produced by Suzhou Newman Analytical Instrument Co., Ltd. (Suzhou, China). The hydrogen atoms inside the samples with different tuff stone powder contents at 7 and 28 days were analyzed to analyze the pore structure. Before the test, the samples were placed in the NEL-VJH concrete intelligent vacuum saturator produced by Beijing Nelder Intelligent Technology Co., Ltd. (Beijing, China). and saturated for 24 h under vacuum with a pressure of −100 kPa. After saturation, NMR was performed on a 20% porosity standard sample.

Scanning electron microscope (SEM) imaging using the ZEISS Gemini SEM500 type instrument, produced by Carl Zeiss (Jena, Germany), was carried out. A small sample of about 5 mm × 5 mm × 3 mm was taken from the damaged tensile specimen and scanned by an electron microscope after gold coating.

The drying shrinkage was determined using the ‘Cement Mortar Dry Shrinkage Test Method’ (JC/T 603-2004) [42]. The specimen was 25 mm × 25 mm × 280 mm. The HZJC-TXJL-059 alkali aggregate specific length meter produced by Tianjin Kexing Instrument and Equipment Co., Ltd. (Tianjin, China). was used to measure the drying shrinkage values of different tuff stone powder contents when they are cured in a dry shrinkage curing box at a temperature of 20 °C ± 3 °C and relative humidity of 50% ± 4% for 3, 7, 14, 21, and 28 days.

## 3. Results, Analysis, and Discussion

### 3.1. Uniaxial Tensile Test

**Figure 4:**
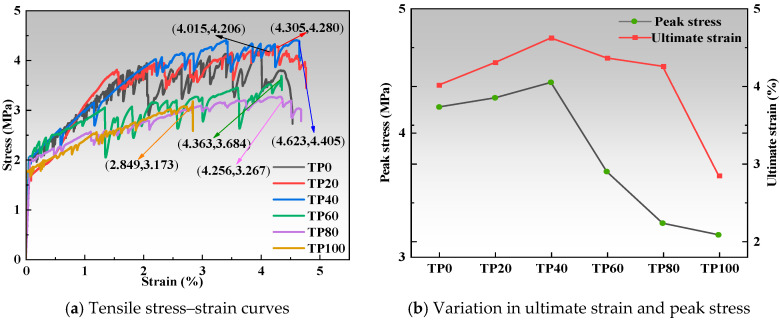
Stress–strain curve.

The tensile stress–strain curves in Figure 4a reveal that each specimen, except for the TP100 specimen, displays characteristic strain hardening and multiple cracking behavior. When the tuff stone powder content is 0%, the ultimate tensile strain and peak tensile stress of TP0 are 4.0% and 4.2 MPa, respectively. When the tuff stone powder content is 20%, the maximum ultimate tensile strain and peak tensile stress of TP20 are 4.3% and 4.3 MPa, respectively, 7.2% and 1.7% higher than the control sample TP0. For 40% tuff stone powder, the ultimate tensile strain and peak tensile stress of TP40 reach the maximum values of 4.6% and 4.4 MPa, i.e., 15.1% and 4.7% higher than the control specimen, respectively. When the tuff stone powder content is 60%, the ultimate tensile strain and peak tensile stress of TP60 are 4.4% and 3.7 MPa, respectively. The ultimate tensile strain is 8.7% higher than that of the control sample, while the peak tensile stress is 12.4% lower than that of the control specimen. When the content of tuff stone powder is 80%, the ultimate tensile strain and peak tensile stress of TP80 are 4.3% and 3.3 MPa, respectively. The ultimate tensile strain is 6% higher than that of the control specimen, while the peak tensile stress is 22.3% lower than that of the control specimen. When the content of tuff stone powder is 100%, the ultimate tensile strain and peak tensile stress of TP100 are 2.8% and 3.2 MPa, respectively, 29% and 24.5% lower than the control specimen. The ultimate tensile strain is less than 3%, which is below the minimum requirements for ECC performance.

In summary, an increase in the dosage of tuff stone powder from 0% to 80% results in an ultimate tensile strain of over 4%, which is greater than the control specimen. However, when the dosage of tuff stone powder exceeds 40%, the maximum peak stress of the ECC begins to decline compared to that of the benchmark specimen. When tuff stone powder completely replaces the fine quartz sand, the ultimate tensile strain and the maximum peak stress obtain the smallest value, respectively. It can be observed that a certain amount of tuff stone powder has a beneficial effect on improving the ECC peak stress and ultimate tensile strain.

Figure 4b shows that the ultimate tensile strain and peak stress change curve, the ultimate tensile strain and peak tensile stress both show an increase, which declines afterward with the increase in the dosage of tuff stone powder. When the replacement ratio of tuff powder is 20–40%, the peak stress and ultimate tensile strain are improved to a certain extent compared with the control specimen. This may be because when the tuff stone powder dosage is low, it has a step-by-step micro-aggregate filling effect to optimize the matrix pore space, making the materials in the ECC matrix and the fibers bonded more tightly, thereby increasing the peak stress and ultimate tensile strain [38]. When the replacement ratio of tuff powder is 60–100%, the peak stress and ultimate tensile strain are reduced to a certain extent compared with the control specimen. This may be because when the tuff stone powder dosage is higher, it leads to over-saturation of the internal pore structure of the concrete and an increase in internal defects, which leads to the instability of the matrix. The pore structure became unfavorable for stress transfer, and the fiber–matrix bonding decreased, leading to reduced peak stress and ultimate tensile strain.

### 3.2. Flexural and Compressive Strengths

**Figure 5:**
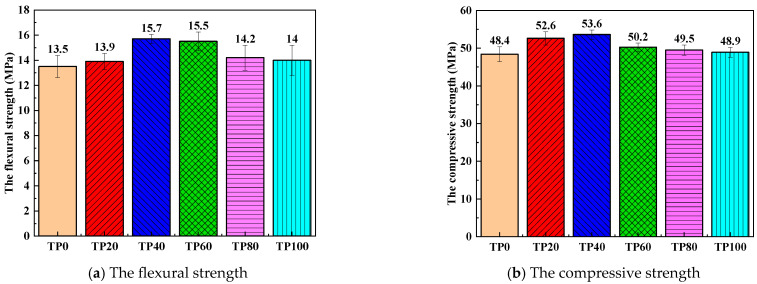
The compressive and the flexural strength of different tuff stone powder content.

Figure 5a,b show the flexural and compressive strength results of the ECC developed over 28 days. When the dosage of tuff stone powder is 0%, the flexural and compressive strengths of TP0 are 13.5 MPa and 48.4 MPa, respectively. For 20% tuff stone powder content, the flexural and compressive strengths of TP20 are 13.9 MPa and 52.6 MPa, respectively, representing a 3% and 8.7% increase over the control ECC specimen PVA-TP0. When the dosage of tuff stone powder is 40%, the flexural and compressive strengths of TP40 are 15.7 MPa and 53.6 MPa, respectively, i.e., 16.3% and 10.7% higher than the control specimen. Similarly, when the dosage of tuff stone powder is 60%, the flexural and compressive strengths of TP60 are 15.5 MPa and 50.2 MPa, respectively, representing an increase of 14.8% and 3.7% compared to the control specimen. Also, when the dosage of tuff stone powder is 80%, the flexural and compressive strengths of TP80 are determined to be 14.2 MPa and 49.5 MPa, respectively, which are 75.2% and 2.3% higher than those of the benchmark specimen. When the dosage of tuff stone powder is 100%, the flexural and compressive strengths of TP100 are 14 MPa and 48.9 MPa, respectively, indicating a 3.7% and 1.0% increase, respectively, compared to the control ECC specimen.

In summary, when the dosage of tuff stone powder increases from 0% to 100%, the flexural and compressive strengths improve. With the increase in the dosage of tuff stone powder, the flexural and compressive strength of each specimen exhibits a trend of increasing and then decreasing, and when the dosage of tuff stone powder is 40%, with the maximal value of flexural and compressive strength is achieved. Thus, incorporating tuff stone powder into ECC is conducive to enhancing its flexural and compressive strengths. This result may be explained by the particle size distribution curve in Figure 2. Tuff stone powder has a smaller particle size than quartz sand, which can better fill the matrix, thereby improving the pore structure of the matrix. Further, the tuff stone powder increases the total binder content in the ECC matrix, and the wrapping of aggregates has been enhanced to a certain extent. At a replacement ratio of 20%, the tuff stone powder dosage is relatively low, and the effect of particle filling is not particularly pronounced. However, as the dosage of tuff stone powder increases, particle filling gradually becomes more pronounced, which enhances the ECC matrix compactness. The filling effect is best when the dosage is 40%. When the replacement ratio of tuff stone powder is greater than 40%, the dosage of excessive fine aggregate reduces the composite matrix skeleton solidity, and the stability of the matrix decreases.

### 3.3. XRD and TG Analyses

**Figure 6:**
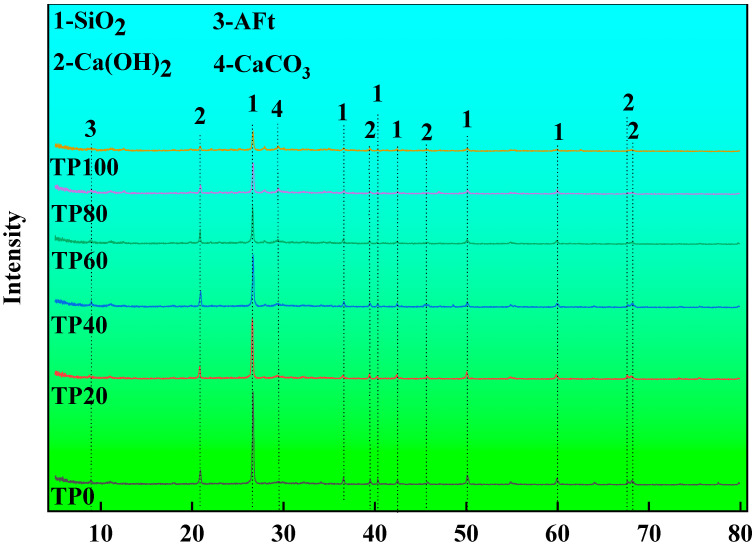
The 28d XRD pattern of different tuff stone powder replacement ratios.

Figure 6 shows the X-ray diffraction (XRD) patterns of 28d-cured ECC specimens for different tuff stone powder replacement ratios. It can be observed that the primary components of ECC are Silica (SiO_2_) and Portlandite (Ca(OH)_2_) after tuff stone powder replaces quartz sand with different replacement ratios, there is also a small amount of ettringite (AFt) and calcite (CaCO_3_) [43,44] The diffraction peak of SiO_2_ is the strongest, and its intensity decreases with an increase in the replacement ratio of tuff stone powder. This is mainly because the main component of quartz sand in ECC is SiO_2_. However, the main component of tuff stone powder is SiO_2_, which is lower than that of quartz sand, so it decreases with the increasing tuff stone powder replacement ratio. When quartz sand is completely replaced by tuff stone powder, the intensity of the SiO_2_ diffraction peak is still high. Ca(OH)_2_ is the hydration product of Portland cement, and its intensity of the diffraction peak decreases gradually with the increase in the tuff stone powder replacement ratio. This is mainly because the tuff stone powder particles provide nucleation sites for the ECC hydration products. With the secondary hydration reaction of the tuff stone powder, the hydration products generated by the partially reactive SiO_2_, Al_2_O_3_, and Ca(OH)_2_ in the fly ash and silica fume closely connect the tuff stone powder and the cement hydration products to fill the pores and consume a part of Ca(OH)_2_ [45,46]. It is seen that the tuff stone powder can improve the cement hydration products, yet its incorporation does not produce new substances.

**Figure 7:**
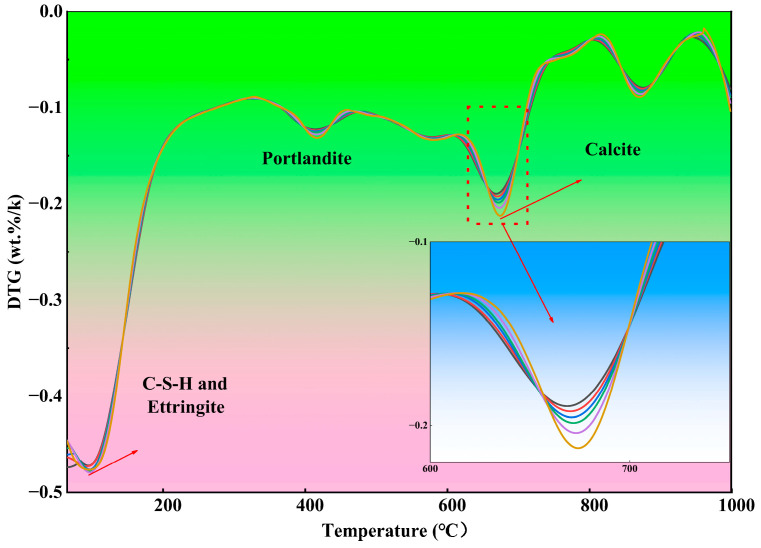
28d TG analysis of different tuff stone powder replacement ratios.

The results of the thermogravimetric analysis (TG) of 28-day cured ECC specimens with different replacement ratios of tuff stone powder are shown in Figure 7. The mass of each specimen decreases with the increase in temperature. At 1000 °C, the mass loss rate of each sample is between 13.36% and 14.13%. When the temperature is 0~200 °C, 400~500 °C, 600~700 °C, and 800~900 °C, the curve reaches the peak value, and the weight loss rate of the sample is accelerated. This is because a series of reactions occur within the matrix as the temperature increases. The peak observed between 600~700 °C, resulting from the decarbonation of calcite, shows an increasing trend with the addition of tuff stone powder, and there is no significant difference between the other three peaks. It is also noted that tuff stone powder can improve cement hydration products [46]. Although the incorporation of tuff powder improves the hydration products in ECC, when the content of tuff powder exceeds 40%, the adverse effect of the reduced matrix strength caused by the supersaturation of pore structure on the performance of ECC is greater than the promotion effect of tuff powder on the hydration products. After the quartz sand is replaced by a large amount of tuff powder, too much fine aggregate in the ECC matrix may lead to matrix instability, so the mechanical properties of ECC are gradually reduced. However, incorporating tuff powder does not cause ECC to produce new chemicals.

### 3.4. Fourier Transform Infra-Red Spectroscopy Test

**Figure 8:**
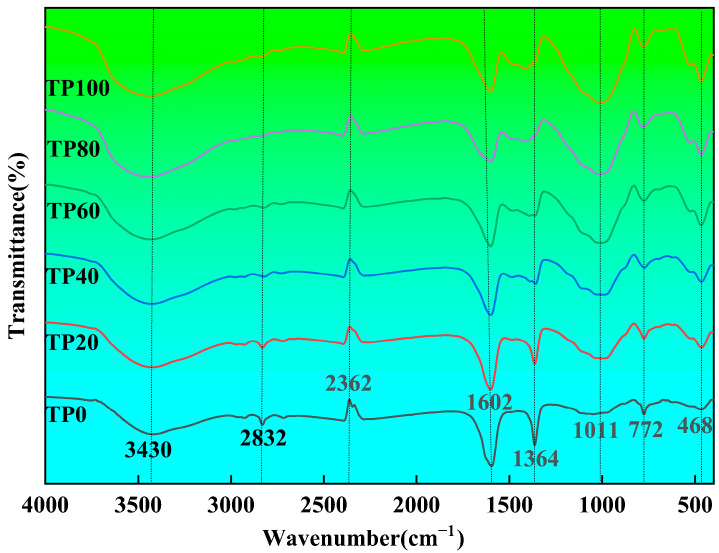
28d FTIR pattern of different tuff stone powder replacement ratios.

Figure 8 shows the FTIR spectra of different tuff stone powder replacement ratios after 28 days of age. The antisymmetric stretching vibration of -O-H corresponds to C-S-H gel, Ca(OH)_2_ and adsorbed water. The symmetrical stretching vibration of -C-H is attributed to the residual organic matter of the water-reducing agent. The stretching vibration of C=C=C is the interference peak of CO_2_ in the test environment. The symmetrical stretching vibration of C=O corresponds to calcite. The antisymmetric bending vibration of -C-H is caused by the residual organic matter of the water-reducing agent. The stretching vibration of C-O corresponds to hydration products such as C-S-H gel and calcium aluminate hydrate. The out-of-plane bending vibration of -C-H is caused by the residual organic matter of the water-reducing agent. The in-plane bending vibration of Si-O corresponds to SiO_2_. It is observed that when the dosage of tuff stone powder is 0, the broad peak of -O-H anti-symmetric telescopic vibration of TP0 appears at 3430 cm^−1^, the symmetric telescopic vibration peak of -C-H, and the anti-symmetric bending vibration peak appeared at 2832 cm^−1^ and 1364 cm^−1^, respectively, while the peak of C=C=C telescopic vibration appeared at 1602 cm^−1^. Also, the symmetric telescopic vibration peak of C=O at 1602 cm^−1^, telescopic vibration peak of C-O at 1011 cm^−1^, out-of-plane bending vibration peak of -C-H at 772 cm^−1^, and in-plane bending vibration peak of Si-O at 468 cm^−1^ were observed. The transmittance of the -C-H and C=O vibration peaks exhibited a decreasing trend with increased tuff stone powder doping. In contrast, the transmittance of the telescopic vibration peak of C-O showed an increasing trend with the increase in tuff stone powder doping. The symmetric telescopic vibration peak of C=O was shifted with the dosage of tuff stone powder. The other characteristics of the peaks, such as transmittance and location, were the same as those of the characteristic peaks of TP0. Therefore, it is inferred that tuff powder incorporation does not lead to the formation of new chemical bonds in ECC. Furthermore, comparison of characteristic peak intensities was conducted through a focus on analyzing the changes in light transmittance at 1011 cm^−1^ (C-O stretching vibration, corresponding to C-S-H gel) and 3430 cm^−1^ (-O-H stretching vibration). The transmittance was the lowest at 1011 cm^−1^ in the TP40 group (18% lower than that of TP0), indicating that the formation of C-S-H gel was the highest. The light transmittance was the highest at 3430 cm^−1^, indicating that the hydroxyl content in the matrix decreased after the zeolite mineral adsorbed free water, which was consistent with the result that the shrinkage increase (17.5%) of the TP40 group in the drying shrinkage test was lower than that of the other groups.

### 3.5. Pore Size and Porosity Test

**Figure 9:**
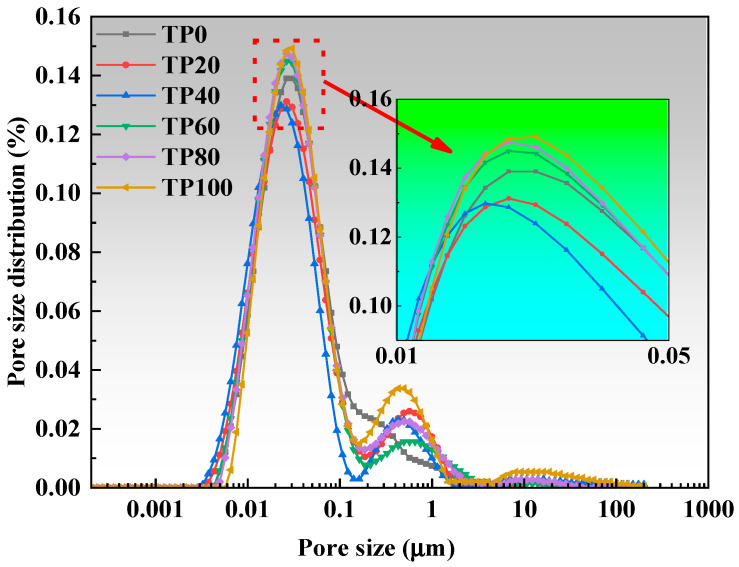
Pore size distribution curves for varying tuff stone powder contents (7d).

**Figure 10:**
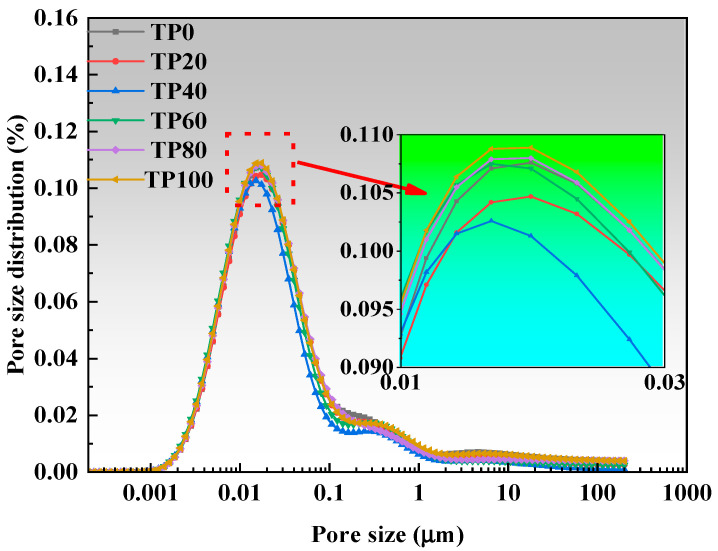
Pore size distribution curves for varying tuff stone powder contents (28d).

Figure 9 and Figure 10 present the porosity and pore size distribution results. As the content of tuff stone powder increases, the pore size of the most probable hole decreases first and then increases. At a tuff stone powder content of 40%, the pore size of the most probable hole reaches a minimum value. Upon reaching 80% tuff stone powder content, the pore size of the most probable hole is similar to that of the benchmark most probable hole. When the content of tuff stone powder reaches 100%, the pore size of the most probable hole reaches the maximum value. This is primarily because the micro-aggregate filling effect is more pronounced when the tuff stone powder content is minimal [47]. When the tuff stone powder content is excessive, the filling is supersaturated, and the excess tuff stone powder exists in the hydration reaction product in a ‘free’ state, forming a large number of pores and destroying the internal pore structure of the matrix [45,48].

**Figure 11:**
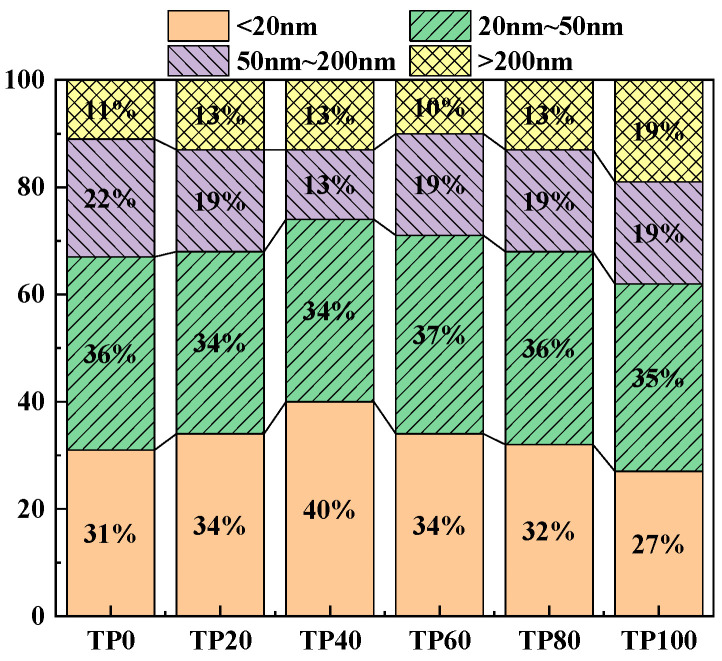
Cumulative proportion of different pore diameters (7d).

**Figure 12:**
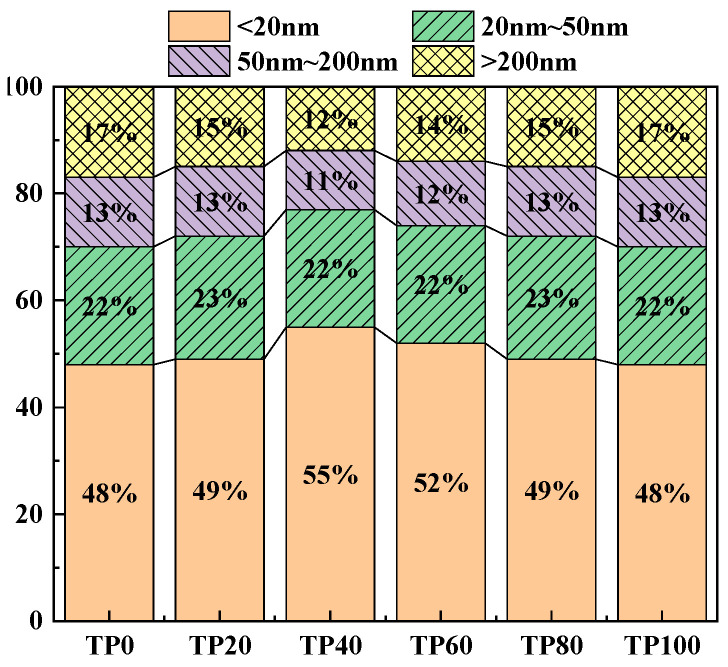
Cumulative proportion of different pore diameters (28d).

**Figure 13:**
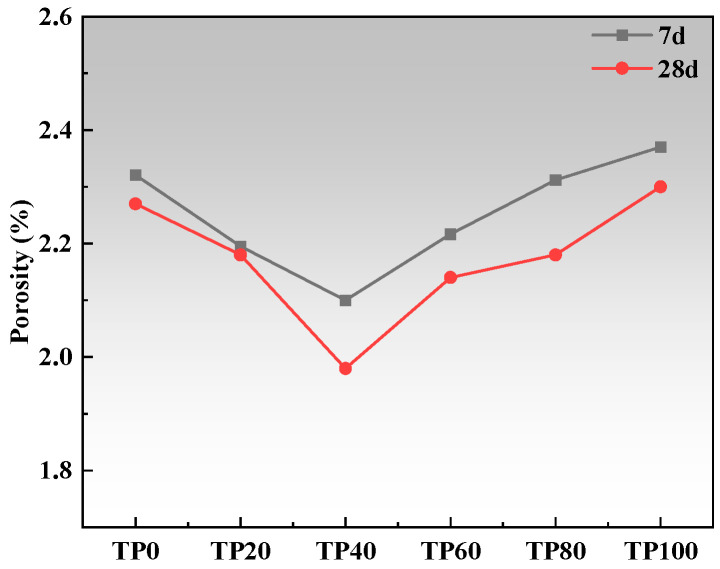
Porosity curves of different tuff stone powder content.

Wu and Lian [49] classified the pores in concrete as harmless pores (<20 nm), less harmful pores (20 nm~50 nm), harmful pores (50 nm~200 nm), and more harmful pores (>200 nm) based on the effects of different pore sizes on concrete properties. Increasing the percentage of harmless and less harmful pores and decreasing the percentage of pores >50 nm can improve the concrete properties. Figure 11 and Figure 12 show the cumulative percentage of pores in ECC at different ages incorporated with varying amounts of tuff stone powder admixtures. It is seen that mainly the pores of sizes <20 nm and > 50 nm have a significant change. When the dosage of tuff stone powder is 0%, the harmless pores below 20 nm in TP0 7 days and 28d accounted for 31% and 48%, and the harmful pores >50 nm accounted for 33% and 30%. When the dosage of tuff stone powder was 20%, the harmless pores <20 nm in TP20 7 days and 28 days accounted for 34% and 49%, which increased by 9.7% and 2.1% compared with the control specimen, the harmful pores above 50 nm accounted for 32% and 28%, respectively, which decreased by 3% and 6.7% compared with the control specimen. When the dosage of tuff stone powder is 40%, the harmless pores <20 nm of TP40 7 days and 28 days accounted for 40% and 55%, which increased by 29% and 14.6% compared with the control specimen. The harmful pores >50 nm accounted for 26% and 23%, which decreased by 21.2% and 23.3% compared with the control specimen, respectively. When the tuff stone powder dosage is 60%, the harmless pores <20 nm of TP60 7 days and 28 days account for 34% and 52%, respectively, which increased by 9.7% and 7.3% compared with the control specimen, the harmful pores >50 nm were 29% and 26%, which decreased by 12.1% and 13.3% compared with the control specimen. When the dosage of tuff stone powder is 80%, the harmless pores <20 nm in TP80 7 days and 28 days account for 32% and 49%, respectively, which are 3.2% and 2.1% higher than the control specimen, the harmful pores >50 nm account for 32% and 28%, which are 3% and 6.7% lower than the control specimen, respectively. When the dosage of tuff stone powder is 100%, the harmless pores <20 nm of TP100 7 days and 28 days account for 27% and 48%, respectively, which increase by 2.9% and 0% compared with the control specimen, the harmful pores >50 nm account for 38% and 30%, which decrease by 15.2% and 0% compared with the control specimen, respectively. The porosity curves at different ages for ECC specimens incorporated with varying tuff stone powder contents (Figure 13) indicate that the porosity of different ages first increases and then decreases with the increase in tuff stone powder content. The porosity at 28-day age is lower than that at 7-day age. The porosity is reduced mainly because the matrix compactness increases with the continuous hydration reaction of the ECC matrix, so the pore structure is persistently improved, and the porosity is reduced. However, the ECC porosity increases when too much tuff stone powder is incorporated, leading to reduced matrix compactness, increased internal defects, and an increased proportion of deleterious porosity. This is detrimental to the mechanical properties of the ECC slurry, signifying a decline in flexural and compressive strength.

### 3.6. Scanning Electron Microscopy (SEM)

**Figure 14:**
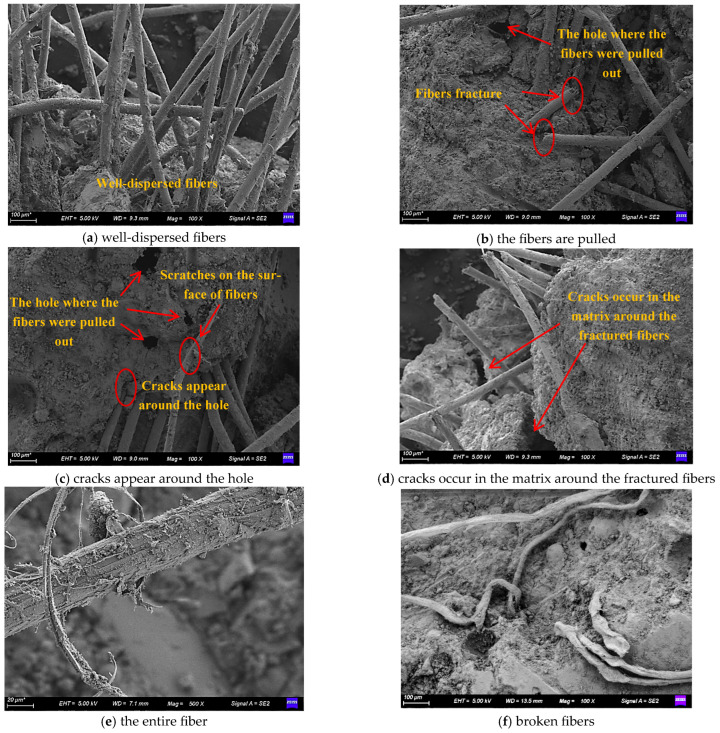
SEM images of the tensile specimen after failure.

Figure 14 shows the SEM imaging of ECC incorporating 40% tuff stone powder. There is no apparent difference in the microscopic morphology of each tensile specimen after failure. Therefore, only the TP40 test results are analyzed. It can be seen from Figure 14a that the ECC prepared with a PVA fiber content of 2.5% has good fiber dispersion, and the vertical and horizontal distribution of the fibers forms a network, which makes the fibers in ECC tightly bonded to the matrix. The fibers are randomly distributed in the matrix, which positively affects the deformation of ECC and plays a role in the fiber-reinforced tensile strain. This is also important for ECC tensile strain to be superior to ordinary concrete. From Figure 14b, it can be seen that there are two forms of fiber failure, i.e., fiber pull-out failure and fiber tensile failure. PVA fiber is a kind of hydrophilic fiber. Physical and mechanical friction bonding and chemical hydrogen bonding exist between the PVA fiber and ECC matrix [50]. Consequently, the bonding between fiber and matrix becomes too strong, and most of the fiber failure modes are tensile failure. Figure 14c,d show that when the ECC specimen is deformed by the load, some of the matrix cracks first. Consequently, the load is borne by the fibers around the matrix due to the fiber-bridging effect. As the load increases, the fibers poorly bonded to the matrix are pulled out to form holes. As the load increases, the stressed fibers that are well-bonded to the matrix begin to thin out until they break. Figure 14e,f show that the fiber is used effectively.

In summary, microcracks are generated in the matrix, and fibers are damaged, indicating that at this strength, the ECC matrix can be fully utilized by all materials to bring out their respective properties, co-ordinate the deformation together, and dissipate the energy due to the external forces on the ECC matrix, which is an essential safeguard for the normal performance of the ECC.

### 3.7. Drying Shrinkage Test

**Figure 15:**
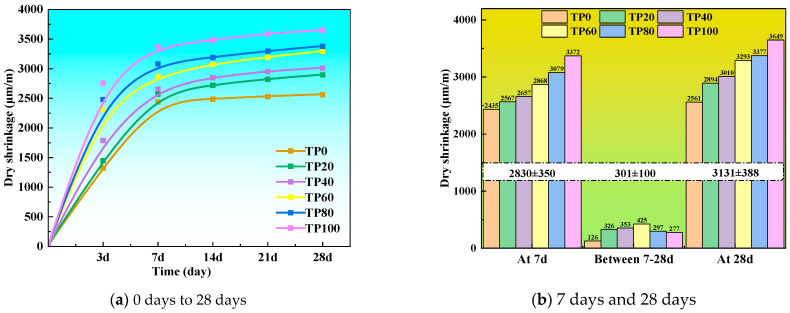
Dry shrinkage values of different tuff stone powder replacement ratios.

Figure 15 shows the drying shrinkage of ECC specimens incorporated with varying dosages of tuff stone powder. When the tuff stone powder content is 0%, the drying shrinkage rates of TP0 at 7 days and 28 days are 2435.3 μm/m and 2561.5 μm/m, respectively. When the content of tuff stone powder is 20%, the drying shrinkage values of TP20 at 7 days and 28 days are 2567.8 μm/m and 2894.5 μm/m, respectively, which are 5.4% and 12.9% higher than those of the control specimen TP0. When the content of tuff stone powder is 40%, the drying shrinkage rates of TP40 at 7 days and 28 days are 2657.0 μm/m and 3010.2 μm/m, respectively, which are 9.1% and 17.5% higher than those of the control specimen. When the content of tuff stone powder is 60%, the drying shrinkage rates of TP60 at 7 days and 28 days are 2868.4 μm/m and 3293.7 μm/m, which are 17.8% and 28.6% higher than those of the control specimen. When the content of tuff stone powder is 80%, the drying shrinkage rates of TP80 at 7 days and 28 days are 3079.7 μm/m and 3377.3 μm/m, respectively, which are 26.5% and 31.8% higher than that of the control specimen. When the content of tuff stone powder is 100%, the drying shrinkage rates of TP100 at 7 days and 28 days are 3372.1 μm/m and 3649.6 μm/m, which are 38.5% and 42.5% higher than that of the control specimen. It can be observed that with the increase in tuff stone powder content and age, the drying shrinkage value of ECC increases gradually. Adding tuff stone powder is not conducive to the drying shrinkage performance of ECC [51].

This result may be because tuff stone powder has a large internal specific surface area due to its lamellar structure, resulting in a part of the free water being adsorbed. Also, adding tuff stone powder is equivalent to increasing the cementitious material, which requires more free water to participate in the reaction. The release of thermal energy will also increase, resulting in accelerated consumption of free water in the pores of the ECC matrix, thereby exacerbating the shrinkage deformation of the matrix, and the drying shrinkage is significantly increased. In addition, when the quartz sand is replaced by tuff stone powder, too much fine aggregate in the ECC matrix leads to instability of the matrix skeleton, making the matrix more prone to shrinkage deformation. In addition, the drying shrinkage of each specimen occurred mainly in the first 7 days, and then the drying shrinkage value stabilized. This may be because the increase in tuff stone powder leads to the acceleration of the hydration reaction. The hydration reaction in the early stage is rapid and tends to be complete, and the hydration in the later stage slows down [52]. In the later stage of hydration, tuff stone powder reacts with Ca(OH)_2_ and other volcanic ash and overlaps with each other to fill ECC to connect pores and optimize pore structure, so the rate of increase in drying shrinkage value slows down [46,53].

### 3.8. Correlation of Multi-Characterization Data

Correlation between mechanical properties and phase changes: Under the dosage of 40% tulimestone powder in the TP40 group, XRD showed that the intensity of the Ca (OH)_2_ peak decreased by 32%, TG showed that the weight loss rate at 100–300 °C (C-S-H dehydration) reached 3.6%, and the corresponding ultimate tensile strain (4.6%) and compressive strength (53.6 MPa) reached their peak. It is confirmed that the dense C-S-H gel generated by secondary hydration is the core for the improvement of mechanical properties.

Correlation between pore structure and shrinkage performance: NMR showed that the proportion of harmless pores (<20 nm) in the TP40 group at 28 days was 55%, and the maximum pore size was < 10 nm. FTIR indicated a decrease in the intensity of the hydroxyl peak at 3430 cm^−1^, which jointly explained the reason why its drying shrinkage rate (3010.2 μm/m) only increased by 17.5% compared with the reference group and was lower than that of granite powder.

## 4. Conclusions

In this paper, the influence of tuff stone powder content on the mechanical properties, X-ray signal, thermogravimetric curve, chemical bond type, pore structure, microstructure, and drying shrinkage of high-ductility polyvinyl alcohol fiber engineered cementitious composites has been investigated. The tuff stone powder was used as a variable instead of quartz sand. The following conclusions are drawn from the results obtained.

(1) When the tuff stone powder content does not exceed 80%, all tensile specimens show good work hardening and multi-crack cracking characteristics, and the tensile elongation at break exceeds 3%, which meets the ECC performance requirements. Tensile elongation at break, peak tensile stress, compressive strength, and flexural strength increase first and then decrease with increasing tuff stone powder addition. The mechanical properties are best when the tuff stone powder content is 40%.

(2) The dosage of tuff stone powder can enhance the ECC hydration products while simultaneously filling the harmful pores within the matrix. As the tuff stone powder content increases, the ECC porosity initially decreases and then increases afterward. The most frequently occurring pore size also exhibits the same trend. The harmless pore size <20 nm and the harmful pore aperture >50 nm vary greatly. The harmful pore aperture is gradually reduced. The 28-day porosity is reduced compared with the 7-day porosity.

(3) When the PVA fiber content is 2.5%, the fibers are well dispersed and tightly bonded to the matrix. Incorporating tuff stone powder into ECC improves strain-hardening characteristics. However, the tuff stone powder negatively influences the drying shrinkage of ECC. The drying shrinkage value increases with an increased tuff stone powder content and age.

## 5. Prospects

(1) Extended research: Conduct compound blending experiments of tuff stone powder and mineral admixtures (such as metakaolin) to explore the mechanism of “zeolite adsorption + volcanic ash activity” synergistic regulation of early shrinkage of ECC.

(2) Engineering application: For the arid regions in Northwest China, long-term durability tests (freeze–thaw resistance, carbonation resistance) of tuff stone powder ECC are carried out to promote its practical application in road repair and hydraulic structures.

(3) Theoretical deepening: By integrating molecular dynamics simulations, the interfacial interaction mechanism between zeolite, chlorite and C-S-H gel in tuff stone powder is revealed, providing theoretical support for the microstructure design of ECC.

## Figures and Tables

**Table 1:** Chemical composition of cement.

CaO	SiO_2_	Al_2_O_3_	Fe_2_O_3_	SO_3_	MgO	K_2_O	Na_2_O
54.415%	26.026%	8.221%	3.731%	3.607%	1.625%	1.032%	0.571%

**Table 2:** Main properties of cement.

Specific Surface Area (m2/kg)	Loss on Ignition (%)	Initial Setting Time (min)	Final Setting Time (min)	Uniaxial Compressive Strength (MPa)		
				3d	7d	28d
352	1.6	127	230	25	36	47.2

**Table 3:** Main properties of fly ash.

Fineness (45 μm Remained)	Sulfur Trioxide	Loss on Ignition	Moisture Content	Water Demand Ratio	Chlorine Ion Content	Strength Activity Index
26.1%	1.48%	4.3%	0.72%	94%	0.012%	80.8%

**Table 4:** Main properties of silica fume.

Silicon Dioxide	Loss on Ignition	Moisture Content	Water Demand Ratio
92.7%	2.3%	0.8%	118%

**Table 5:** XRF analysis of tuff stone powder.

SiO_2_	Al_2_O_3_	Fe_2_O_3_	CaO	MgO	Na_2_O	K_2_O	TiO_2_
47.4%	18.6%	12.3%	12.2%	4.88%	1.47%	1.19%	0.87%

**Table 6:** XRD analysis of tuff stone powder.

Zeolite	Chlorite	Feldspar	Quartz	Magnetite	Mica
52.8%	18.4%	18.0%	5.9%	2.7%	2.2%

**Table 7:** Fiber properties.

Length (mm)	Diameter (μm)	Density (g/cm^3^)	Tensile Strength (MPa)	Elongation (%)	Elasticity Modulus (GPa)
12	40	1.30	1560	6.5	41

**Table 8:** ECC mix proportion.

Group	Cement (kg/m^3^)	Fly Ash (kg/m^3^)	Silica Fume (kg/m^3^)	Fine Aggregate		PVA (vol%)	Superplasticizer (wt‰)
				Quartz Sand (%)	Tuff Stone Powder (%)		
TP0	455.5	364.4	91	100	0	2.5	6.59
TP20				80	20		6.80
TP40				60	40		6.97
TP60				40	60		7.11
TP80				20	80		7.22
TP100				0	100		7.32

PVA (vol%)—PVA fiber volume/ECC volume, Superplasticizer (wt‰)—Superplasticizer weight/cementitious material weight.

## Data Availability

The original contributions presented in this study are included in the article. Further inquiries can be directed to the corresponding author.
